# Septic cavernous sinus thrombosis presenting as acute cerebral infarction and aneurysmal subarachnoid hemorrhage: Case report

**DOI:** 10.1097/MD.0000000000036123

**Published:** 2023-11-24

**Authors:** Pengchen He, Zongping Li, Han Jiang

**Affiliations:** a Department of Neurosurgery, Mianyang Central Hospital Affiliated to University of Electronic Science and Technology of China, Mianyang, China; b Department of Rehabilitation Therapy, Mianyang Central Hospital Affiliated to University of Electronic Science and Technology of China, Mianyang, China.

**Keywords:** acute cerebral infarction, aneurysmal subarachnoid hemorrhage, case report, septic cavernous sinus thrombosis

## Abstract

**Rationale:**

Septic cavernous sinus thrombosis (SCST) is a rare infectious thrombophlebitic disease. The infection often arises from the tissues surrounding the cavernous sinus as well as the cavernous sinus drainage. Early symptoms of SCST include fever, headache, proptosis, ptosis, bulbar conjunctival edema, and limited eye movement. The complications include venous sinus thrombosis, intracerebral abscess, and subdural empyema. Aneurysmal subarachnoid hemorrhage combined with acute cerebral infarction has not been reported.

**Patient concerns:**

A 46-year-old man presented with visual impairment in his right eye and intermittent headache for 2 months. Ten days later, the patient developed a sudden loss of consciousness, coma, cardiac arrest, and respiratory arrest. The patient eventually died.

**Diagnoses:**

SCST, acute cerebral infarction, aneurysmal subarachnoid hemorrhage, anterior cerebral artery aneurysm.

**Interventions:**

Antiplatelet and lipid-lowering therapy, antibiotic treatment, emergency aneurysm clipping, and decompressive craniectomy.

**Outcomes:**

The patient underwent emergency aneurysm clipping and decompressive craniectomy, and postoperative head computed tomography showed a massive cerebral infarction in the right cerebral hemisphere. The patient eventually died.

**Lessons:**

We report a case of SCST mainly presenting as acute cerebral infarction and aneurysmal subarachnoid hemorrhage, with an acute onset and ultimately a poor prognosis. This complication is extremely rare and have not yet reported according existing literatures but can be life-threatening if not recognized and treated promptly. Early antibiotic administration and early sinus drainage may alter the patient’s prognosis. By describing this unusual the case we hope to raise awareness of the need of early illness detection and treatment in order to avoid catastrophic consequences. It also exemplifies the mechanism of acute inflammatory disorders and aneurysm development.

## 1. Introduction

Septic cavernous sinus thrombosis (SCST) is a rare infectious thrombophlebitic disease. The infection often arises from the tissues surrounding the cavernous sinus (CS) as well as the CS drainage. Besides, sinus infection is also one of the most common sources of infection.^[1,2]^ Even with the widespread use of antibiotics and continued technological advances in imaging techniques, the SCST mortality rate is still roughly 14% to 30%.^[3]^ The anatomy of the CS determines the clinical manifestation of the SCST. Early symptoms of SCST include fever, headache, proptosis, ptosis, bulbar conjunctival edema, and limited eye movement.^[4]^ Imaging examinations often show filling defects in the CS region, lateral displacement of the lateral wall of the CS, and soft tissue masses in the CS region.^[5]^ Different complications of SCST often determine the prognosis of the disease. The complications include venous sinus thrombosis, intracerebral abscess, and subdural empyema.^[2,5]^ However, aneurysmal subarachnoid hemorrhage combined with acute cerebral infarction has not been reported.

## 2. Case report

**Figure 1.**
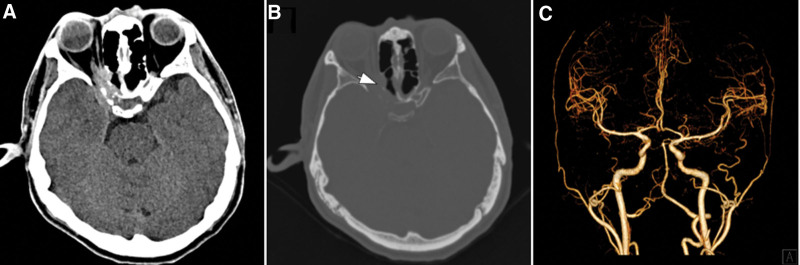
(A) Head CT indicated abnormal signal shadows in the right paranasal sinus considering the possibility of paranasal sinusitis or malignant tumor on first admission. Right posterior ethmoid sinus with bone was destructed in the corresponding areas(arrow indicates). (C) CT angiography indicated no aneurysms or vascular malformations were found.

**Figure 2.**
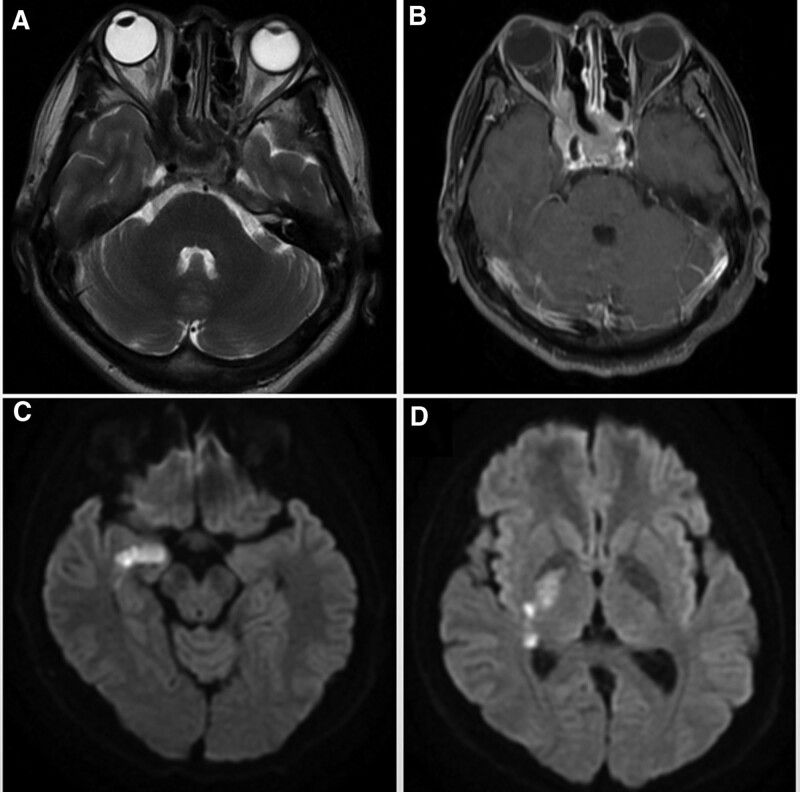
(A) MR T2-weighted image showed low signal shadow can be seen in the paranasal sinus and right cavernous sinus. (B) Enhanced MR T1-weighted image showed the lesions were seen occupying the cavernous sinuses, sphenoid sinuses, and part of paranasal sinuses and entering the optic canal, which appears as internal carotid artery enhancement. (C and D) Diffusion-weighted image showed high signal shadow in the right hippocampus and basal ganglia region, suggesting acute cerebral infarction.

**Figure 3.**
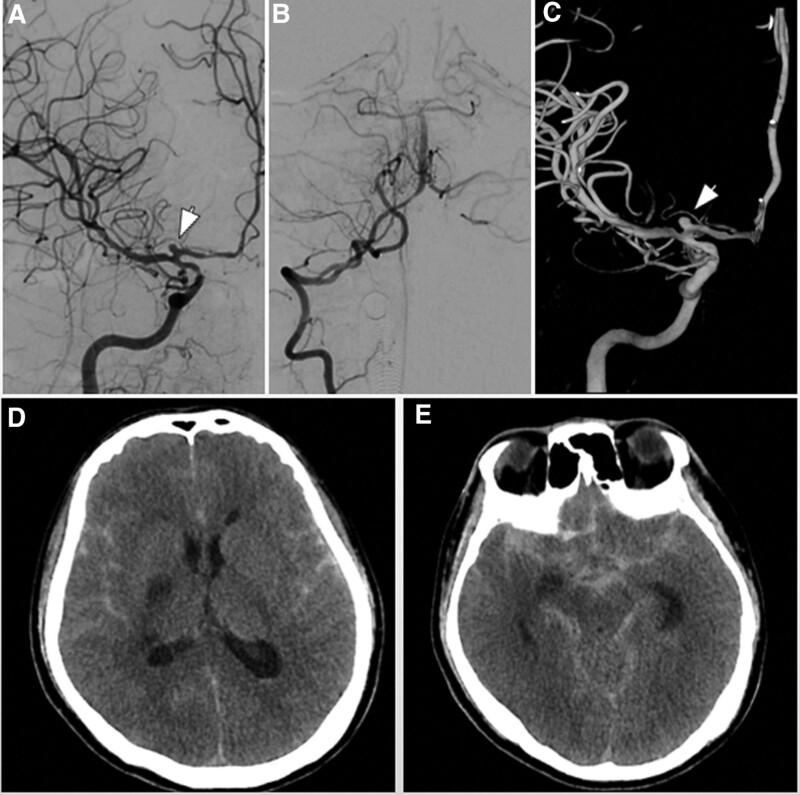
(A–C) Cerebral digital subtraction angiography showed a cystic aneurysm in the A1 segment of the right anterior cerebral artery with a size of approximately 2.8 × 2.9 mm and a neck of approximately 2.3 mm. (D–E) Repeat head CT showed extensive subarachnoid hemorrhage with intraventricular hemorrhage.

**Figure 4.**
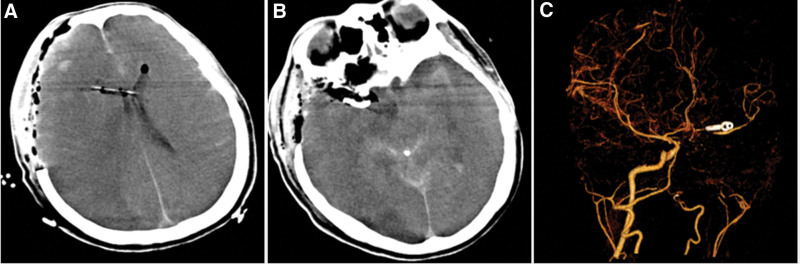
(A and B) Repeat head CT showed massive cerebral infarction in the right cerebral hemisphere after aneurysm clipping surgery. (C) CTA showed right internal carotid artery was disappeared, suggesting occlusion completely.

A 46-year-old man was admitted to the hospital with blurred vision and headache for 2 months. The patient had no other chronic diseases or immunodeficiency disorders. Neurological examination showed decreased visual acuity and delayed pupillary light reflex in his right eye, with no limited eye movement or ptosis. Blood analysis revealed mildly elevated levels of infectious markers. Head computed tomography (CT) and CT angiography (CTA) indicated paranasal sinusitis or malignant tumor of the right sphenoid sinus, involving the right posterior ethmoid sinus with bone destruction in the corresponding areas. No aneurysms or vascular malformations were found in cranial CTA (Fig. 1). After admission, the patient was prepared for transnasal exploration of the sphenoid cavity and treated with the antibiotic ceftriaxone. One day later, the patient experienced a sudden aggravation of headache with nausea and vomiting. He also developed a fever. The muscle strength of the left upper extremity was grade 4/5, with numbness and hypesthesia of the left extremities. A repeat examination showed significantly elevated levels of infectious markers, and head magnetic resonance imaging (MRI) revealed acute cerebral infarction in the right hippocampus and basal ganglia region. Abnormal signal shadows were seen in the right sinus and CS, local bone thinning, and right optic nerve compression. (Fig. 2). Combined with the neurologist’s advice, the patient was then given antiplatelet and lipid-lowering therapy, with continued antibiotic treatment, and the sphenoid sinus exploration surgery was postponed. Ten days later, the patient developed a sudden loss of consciousness and coma, accompanied by cardiac arrest and respiratory arrest. Cardiopulmonary resuscitation was immediately performed, with tracheal intubation and administration of epinephrine and atropine, and the patient was then transferred to the intensive care unit for further resuscitation treatment. The neurological examination showed that the patient was in a coma, with a right pupil size of 6 mm and left pupil size of 3 mm, and pupillary light reflexes on both sides were absent. Spontaneous breathing was present. Repeat head CT showed extensive subarachnoid hemorrhage with intraventricular hemorrhage. With stable vital signs, cerebral digital subtraction angiography was performed, which showed a cystic aneurysm in the A1 segment of the right anterior cerebral artery with a size of approximately 2.8 × 2.9 mm and a neck of approximately 2.3 mm. A filling defect was observed in the right vertebral artery with a stenosis of approximately 40% (Fig. 3). The patient then underwent an emergency craniotomy and aneurysm clipping, and the aneurysm was completely clamped with a straight 5-mm aneurysm clip. During the surgery, the aneurysm was found to have a thin wall, no intra-aneurysm thrombus, and no calcified plaque in the parent artery. The surgery was successfully completed. However, repeat head CT and CTA showed occlusion of the right internal carotid artery and massive cerebral infarction in the right cerebral hemisphere (Fig. 4). After aggressive resuscitation, the patient’s prognosis was poor, and he was eventually declared clinically dead.

## 3. Discussion

Even with the continuous improvement of modern diagnosis and treatment techniques, SCST is still a rare and highly lethal intracranial infectious disease that should be treated as soon as it is diagnosed.^[6]^ Early diagnosis and treatment are the key to SCST treatment, although intracranial complications are often present at the time of diagnosis due to atypical clinical symptoms in the early phase of the disease.^[5]^

The CS is a paired dural venous sinus in the middle cranial fossa on either side of the sella turcica of the sphenoidal bone. Physiologically, it has many inflows because of connections with the surrounding venous network including the ophthalmic veins, sphenoparietal sinus, superficial middle cerebral vein and pterygoid plexus located in the infratemporal fossa. Its lateral wall consists of dura, and the oculomotor, trochlear, ophthalmic, and maxillary nerves lie within it. Medially, the space is bordered by the cavernous part of the internal carotid artery.^[7,8]^ Theoretically, any infectious lesion in the area where the veins drain into the CS has the potential to cause SCST. SCST caused by infratemporal fossa infection and periorbital cellulitis has been reported.^[3]^ Because the CS is adjacent to the paranasal sinuses, such as the sphenoid sinus, the most common etiology of SCST is paranasal sinus infection.^[1]^ In addition to infection caused by venous drainage, direct infiltration of the infection into the CS is also a main cause. We believe that the patient in this case may cause SCST after drainage of the CS through inflammation of the paranasal sinus, and the internal carotid artery in the CS may be infiltrated by inflammation, leading to the destruction of the internal carotid artery intima. Tissue factors are exposed to the blood, which play a role in coagulation through the coagulation function mechanism, and lead to thrombosis. Meanwhile, this case of SCST was also probably caused by the direct invasion of the CS after destruction of adjacent bone by the sphenoid sinus infection.

Stroke, which is often caused by the acute occlusion of blood vessels, is the leading cause of disability and the second leading cause of death worldwide. The main causes include atherosclerosis, cardiogenic embolism, and small vessel disease.^[9]^ Acute cerebral infarction caused by SCST is extremely rare, and its pathogenesis has not been fully elucidated. It has been suggested^[1]^ that local compression and stenosis of the internal carotid artery leads to increased local blood flow velocity and local vortex formation, which results in thrombosis, thereby leading to the formation of acute infarct foci by blockage of distal branches. In this case, head CTA showed that the affected internal carotid artery did have stenosis, which also supports this view. We also believe that the incompleteness of the sphenoid sinus contributed to the progression of the course of the disease. The head CT revealed that the lateral wall of the sphenoid sinus had signs of bone destruction.

Intracranial aneurysm is a cerebrovascular disease, and the mechanisms of aneurysm formation, growth, and rupture are still being explored. According to the existing literature, the development of intracranial aneurysm is a progressive process influenced by genetic, environmental, and hemodynamic factors. In this case, the intracranial aneurysm was not detected on head CTA after admission. After more than 10 days of treatment, the patient suddenly developed a subarachnoid hemorrhage, which was confirmed by CTA to be caused by a ruptured aneurysm. We consider that due to the inflammatory invasion of the CS, the internal carotid artery intima was infiltrated by inflammation to form infectious arterial vasculitis, thus resulting in a purulent intracranial aneurysm. Infectious vasculitis is caused by infection with infectious agents (bacteria, viruses, or fungi) and leads to structural changes in the intima, resulting in vascular occlusion, ischemia. Local blood vessels lose compliance, and the blood vessel wall is continuously scour by blood to form inflammatory aneurysms.^[10]^ It has been reported that^[11]^ high-resolution MRI can detect lesions of the tunica intima. However, this case progressed rapidly, and it was unfortunate that we did not have the opportunity to perform this examination.

The clinical symptoms of SCST are atypical. SCST often needs to be differentiated from pituitary apoplexy, carotid cavernous fistula, and periorbital cellulitis.^[12]^ The most common symptoms of SCST are periorbital edema and proptosis, followed by headache and cranial nerve palsies (III, VI, V1, and V2). Other clinical signs of SCST include bilateral progressive ocular symptoms, sepsis, altered state of consciousness, loss of vision, and dilated retinal veins.^[3,13]^ Further expansion of infection to adjacent structures may result in loss of vision, hypopituitarism, meningitis, brain abscess, epidural and subdural abscesses, and eventually coma and death.^[14]^ The case we reported first presented with headache and visual impairment, indicating involvement of the optic nerve, along with symptoms of mild cranial nerve palsies, which was consistent with the existing literature. However, symptoms eventually progressed to consciousness disturbance and coma, and the patient was diagnosed with intracranial aneurysm rupture and hemorrhage, which had not been reported in the existing literature. In this instance, the cause of the aneurysm is only conjectural. It is still unknown what causes aneurysm development because there are not enough cases or pathological data. The mechanism of acute inflammation and intracranial aneurysm remains to be further explored.

The consensus for the treatment of SCST is early sinus drainage and broad-spectrum antibiotics, but using anticoagulation remains controversial.^[15]^ It is unknown if the aneurysm rupture that occurred later in this case was caused by the antiplatelet medication that was administered earlier. The use of antibiotics must be based on the pathogenic test results of a blood sample, and third- or fourth-generation cephalosporins may be used empirically if a pathogenic test result is not available. Early debridement and drainage are also critical. When we started treating this patient, we didn’t recognize the illness as a significant infectious disease and instead used antiplatelet medication and low-generation antibiotics without realizing how bad the condition was. Meanwhile, our first course of treatment was interrupted by the unexpected stroke incident. Early drainage can relieve local cranial nerve compression and improve symptoms. At the same time, etiological specimens can be obtained and information on sensitive antibiotics can be obtained. It could eventually alter the course of the disease and improve patient outcomes. Although we performed timely intracranial aneurysm clipping and decompressive craniectomy, the patient had internal carotid artery stenosis and eventually progressed to massive cerebral infarction with a poor prognosis.

## 4. Conclusion

We report a rare case of SCST caused by sinusitis, complicated with severe intracranial vascular disease. Both diagnosis and therapy are difficult and full with pitfalls. The initial clinical symptoms were not severe, and the diagnosis was delayed due to the complications of the disease. This case is worth drawing lessons from, and a joint multidisciplinary discussion on such rare cases may be helpful.

## Author contributions

**Conceptualization:** Han Jiang.

**Data curation:** Han Jiang.

**Formal analysis:** Han Jiang.

**Methodology:** Zongping Li.

**Supervision:** Pengchen He, Han Jiang, Zongping Li.

**Visualization:** Zongping Li.

**Writing – original draft:** Pengchen He.

**Writing – review & editing:** Pengchen He.
